# Innovative Imaging Techniques: Nice to Have or Essential to Treat Complex Diseases?

**DOI:** 10.3390/diagnostics13193127

**Published:** 2023-10-05

**Authors:** Jean-Paul P. M. de Vries

**Affiliations:** Department of Surgery, Division of Vascular Surgery, University Medical Centre Groningen, 9713 GZ Groningen, The Netherlands; j.p.p.m.de.vries@umcg.nl

Nowadays, a substantial number of our patients have multimorbidity, and need treatment for complex diseases. New imaging tools may be of help during all phases of the care process (diagnostic, therapeutic and follow-up), improving clinical outcomes. These imaging techniques may also facilitate a sustainable environment for physicians to work in, with minimal burden on patients, healthcare workers and society. 

In this Special Issue, three out of eight manuscripts focus on diagnostic tools related to abdominal aortic aneurysms (AAAs). Slijkhuis and coworkers determined the value of ultrasound (US) for the detection of inflammatory AAAs (iAAAs) [1]. So far, the golden standard for detecting an iAAA is computed tomography (CT), which needs radiation and may lead to contrast-induced renal insufficiency. In a retrospective validation study the authors proved that ultrasound could rule out iAAAs in all patients negative for iAAA, where a specificity of 98.7% was reached. Only in cases of positive US findings may the more expensive CT technique still be necessary to guide diagnostic microbiological punctures or to plan surgical interventions. On the other hand, two research groups showed that standard CT angiography (CTA) alone does not suffice when attempting to understand the dynamics of the abdominal aorta that includes implanted stent grafts. In their retrospective series, Simmering and coworkers used electrocardiogram (ECG)-gated CTA to determine the geometry changes in two different types of iliac branch devices for the treatment of common iliac artery aneurysms [2]. In addition to the iliac arteries, ECG-gated CTA may be of great help to study the geometry changes in thoracic and abdominal aortic endografts during the cardiac cycle, their influence on cardiac function, as well as the (changed) compliance of the thoraco-abdominal aorta post endovascular repair of aortic aneurysms (EVAR). One of the major complications post EVAR is the occurrence of endoleaks, which repressurise the aorta and may lead to aneurysm growth and even rupture. Endoleaks may be hard to determine even with the use of multiphase CTA. The research group of Fioole showed that dynamic CTA is of added value regarding the detection and classification of slow-flow endoleaks, and especially of type 2 endoleaks which usually originate from backbleeding lumbar arteries [3]. Improvement of the endoleak classification also allows the endovascular specialist to make targeted reinterventions. 

Two manuscripts in this Special Issue are dedicated to patients suffering from peripheral arterial disease (PAD) [4,5]. Ma and coworkers showed how innovative imaging techniques can be used to facilitate frail patients [5]. They performed tissue perfusion measurements in PAD patients after endovascular revascularisation of the lower limb. In a pilot study, significant differences were detected 1 week post revascularisation between patients with and without clinical improvement. The most important conclusion of this small prospective series is the fact that these measurements were safely performed at the patients’ homes without the need to visit the hospital. 

Using modern imaging techniques may lead to improvements in decision making. A good example is the retrospective study by Leemhuis and coworkers in this Special Issue [6]. In a Dutch nationwide cross-sectional survey the authors explored whether experienced pelvic surgeons would change their surgical approach for both anterior- and posterior-column acetabular fractures based on 3D virtual reconstructions versus conventional 2D imaging. The additional 3D information led to a significant change in surgical approach, and the surgeons’ level of confidence increased from 37% to 50%. Due to the retrospective character of the study, no conclusions could be made concerning clinical outcomes. These findings contrast with the added value of imaging modalities used to diagnose neurogenic thoracic outlet syndrome (NTOS). In this Special Issue, Teijink et al. conclude that a specific diagnostic tool to prove NTOS is lacking [7]. Imaging techniques are used to exclude other diagnoses, whereas clinical pattern recognition combined with physical examination is still the golden standard. 

Although standard techniques such as ultrasound, CT and magnetic resonance imaging (MRI) are of great importance for detecting structural abnormalities, these methods are of limited value in assessing changes that occur at the molecular and functional levels. Applications like positron emission tomography (PET)/CT and PET/MRI enable the complementary assessment of molecular changes that precede structural abnormalities. Therefore, hybrid imaging with PET can detect the disease in its earliest stages that are more amenable to interventions, thereby preventing it from becoming an irreversible structural abnormality. In this Special Issue, no studies regarding PET/CT or PET/MRI are included, which is a limitation.

Before implementing (the pilot and transition phase) a new technique, thorough testing and validation should be performed. A good example of this in this Special Issue is the phantom model study by Sikkenk and coworkers, which tested the detection of specific tumour tracers with commercially available laparoscopic surgical systems [8]. Recently, the research group of Suh concluded that the adaptation of new surgical techniques fundamentally necessitates other requirements than just medication or device implementation [9]. The process of the successful implementation of new techniques may be arduous and includes different important phases (discovery, preparation, pilot and transition). The common thread in all of these phases is the need to ensure safety and efficacy of the new surgical or procedural techniques. Parmar and coworkers systematically reviewed the possible facilitators and barriers for the implementation of healthcare innovations [10]. Important facilitators include a balanced composition of implementation teams, alignment with clinical roles, flexible training and organisational support. Well-known barriers include a lack of information, underdeveloped implementation plans, insufficient training and hurdles within the organisation. Despite all worldwide efforts, no single optimal strategy has yet been identified that can guarantee a promising scientific idea will lead to a sustained and widely implemented new (surgical or imaging) technique. In other words, we have to take into account that a substantial number of current medical innovations may fail in the conceptual phase and will remain as “nice-to-have”.
